# Impact of body mass index and diabetes on myocardial fat content, interstitial fibrosis and function

**DOI:** 10.1007/s10554-022-02723-8

**Published:** 2022-10-28

**Authors:** Xin Dong, Mark Strudwick, William YS Wang, Barry A. Borlaug, Rob J van der Geest, Austin CC Ng, Victoria Delgado, Jeroen J. Bax, Arnold CT Ng

**Affiliations:** 1 School of Exercise and Nutrition Sciences, Queensland University of Technology, Brisbane, Australia; 2 Centre for Advanced Imaging, The University of Queensland, Queensland, Australia; 3 Department of Cardiology, Princess Alexandra Hospital, Woolloongabba, QLD Australia; 4 Department of Cardiovascular Medicine, Mayo Clinic, Rochester, MN USA; 5 Department of Radiology, Leiden University Medical Center, Leiden, The Netherlands; 6 Department of Cardiology, Concord Hospital, The University of Sydney, Concord, NSW Australia; 7 Department of Cardiology, Leiden University Medical Centre, Leiden, The Netherlands; 8 Faculty of Medicine, South Western Sydney Clinical School, The University of New South Wales, Warwick Farm, Australia; 9 Department of Cardiology, Leiden University Medical Center, Albinusdreef 2, 2333 ZA Leiden, The Netherlands

**Keywords:** Steatosis, T1 mapping, Speckle tracking, Diabetes, Obesity

## Abstract

**Purpose:**

We hypothesize that both increased myocardial steatosis and interstitial fibrosis contributes to subclinical myocardial dysfunction in patients with increased body mass index and diabetes mellitus.

**Background:**

Increased body weight and diabetes mellitus are both individually associated with a higher incidence of heart failure with preserved ejection fraction. However, it is unclear how increased myocardial steatosis and interstitial fibrosis interact to influence myocardial composition and function.

**Methods:**

A total of 100 subjects (27 healthy lean volunteers, 21 healthy but overweight volunteers, and 52 asymptomatic overweight patients with diabetes) were prospectively recruited to measure left ventricular (LV) myocardial steatosis (LV-myoFat) and interstitial fibrosis (by extracellular volume [ECV]) using magnetic resonance imaging, and then used to determine their combined impact on LV global longitudinal strain (GLS) analysis by 2-dimensional (2D) speckle tracking echocardiography on the same day.

**Results:**

On multivariable analysis, both increased body mass index and diabetes were independently associated with increased LV-myoFat. In turn, increased LV-myoFat was independently associated with increased LV ECV. Both increased LV-myoFat and LV ECV were independently associated with impaired 2D LV GLS.

**Conclusion:**

Patients with increased body weight and patients with diabetes display excessive myocardial steatosis, which is related to a greater burden of myocardial interstitial fibrosis. LV myocardial contractile function was determined by both the extent of myocardial steatosis and interstitial fibrosis, and was independent of increasing age. Further study is warranted to determine how weight loss and improved diabetes management can improve myocardial composition and function.

**Supplementary Information:**

The online version contains supplementary material available at 10.1007/s10554-022-02723-8.

## Introduction

There is a worldwide growing epidemic of obesity and diabetes. Both conditions are associated with mitochondrial dysfunction [1], myocardial steatosis [2], increased interstitial fibrosis [3], and coronary artery disease [4]. Obese and diabetic patients with these changes are more likely to have impaired left ventricular (LV) global longitudinal strain (GLS) and can develop heart failure with preserved ejection fraction (HFpEF) [5, 6]. Recently, we demonstrated that diabetes and increasing body mass index (BMI) have an additive detrimental effect on LV-GLS [7]. However, the pathophysiological interactions between increased BMI, diabetes, myocardial steatosis, interstitial fibrosis, and LV-GLS are unclear.

Cardiac magnetic resonance imaging (MRI) technique such as water and fat separated imaging with variable projection (VARPRO) to calculate myocardial fat (LV-myoFat) content can quantify myocardial steatosis [2]. Similarly, T1 mapping to calculate extracellular volume (ECV) can also quantify the burden of interstitial fibrosis [8]. Finally, echocardiographic LV-GLS can detect subclinical myocardial dysfunction despite normal LV ejection fraction (EF) [7].

Therefore, the present study aimed to evaluate the pathophysiological mechanisms linking myocardial steatosis and interstitial fibrosis secondary to increased BMI and diabetes with LV-GLS. We hypothesize that: (1) increased BMI and diabetes are independently associated with increased myocardial steatosis; (2) increased myocardial steatosis is independently associated with increased myocardial interstitial fibrosis; and (3) both increased myocardial steatosis and interstitial fibrosis are independently associated with LV-GLS.

## Method

### Study population and study protocol

100 volunteers randomly recruited from the community were divided into 3 groups:

- Group 1 included 27 healthy volunteers with normal BMI (< 25.0kg/m^2^);
- Group 2 included 21 healthy volunteers with increased BMI (≥ 25.0kg/m^2^);
- Group 3 included 52 asymptomatic volunteers with type 2 diabetes.

#### Exclusion criteria

Exclusion criteria included age < 18 years, pregnancy, rhythm other than sinus rhythm, known underlying severe coronary artery disease, previous myocardial infarction, LVEF < 50%, moderate/severe valvular stenosis or regurgitation, pre-existing hepatic or renal disorders (i.e., eGFR < 60mL/min/1.73m^2^ by Modification of Diet in Renal Disease [MDRD] equation), inability to undergo a cardiac MRI, and inadequate echocardiographic images for speckle tracking analysis.

For healthy volunteers in Groups 1 and 2, additional exclusion criteria included any underlying cardiovascular risk factors including hypertension, diabetes, active smoking, and current use of any regular medications.

For diabetic volunteers in Group 3, myocardial ischemia as a potential confounding factor was excluded by a normal MRI myocardial adenosine stress perfusion, and absence of macroscopic scar on delayed enhancement. Patients utilizing thiazolidinediones or sodium-glucose co-transporter 2 inhibitors were also excluded as they can affect myocardial steatosis and change myocardial energy substrate usage respectively [9, 10].

#### Study protocol

All subjects’ blood tests, echocardiograms and MRI examinations were performed on the same day after an overnight fast of at least 8h.

Cardiac MRI was used to quantify LV volumes, LVEF and LV mass. Myocardial steatosis was quantified using VARPRO and interstitial fibrosis by ECV as previously published. [3, 8]. Speckle tracking echocardiography was used to quantify LV-GLS.

The study was approved by the institutional ethics committee. All subjects provided written informed consent.

### Demographic, anthropomorphic and metabolic data

Blood pressure (BP) was measured at the time of echocardiography. As LV-GLS is load dependent and correlated with both heart rate and BP, rate pressure product ([RPP], which had higher correlation coefficient with LV-GLS compared to heart rate and BP) was therefore used in the multivariable models. RPP was calculated as the heart rate multiplied by systolic BP during echocardiography.

Blood samples were collected to measure hemotocrit, fasting lipid profile, plasma glucose, glycated hemoglobin (HbA1c), and insulin (see Supplemental Appendix). The homeostatic model assessment index of insulin resistance (HOMA-IR) was computed using the HOMA calculator that utilizes the HOMA2 model [11].

### Cardiac magnetic resonance imaging

Cardiac MRI examinations were performed using a 1.5T Siemens Magnetom Avanto system (Erlangen, Germany). See Supplemental Appendix for cine imaging parameters. LV end-diastolic mass, LV end-diastolic volume (EDV) and LV end-systolic volume (ESV) were measured, and LVEF was calculated. Images were analyzed using MASS V2010-EXP (LUMC, Leiden, The Netherlands). We have previously published the intra- and interobserver measurement variabilities for LV-myoFat and ECV [7].

#### MRI quantification of myocardial fat content

We quantified both the proportion (i.e., LV-myoFat fat fraction) and the total amount of LV myocardial steatosis (i.e., total LV-myoFat volume). Our group has previously validated LV-myoFat fraction by VARPRO against the gold standard proton spectroscopy [2]. See Supplemental Appendix for VARPRO imaging parameters.

**Fig. 1:**
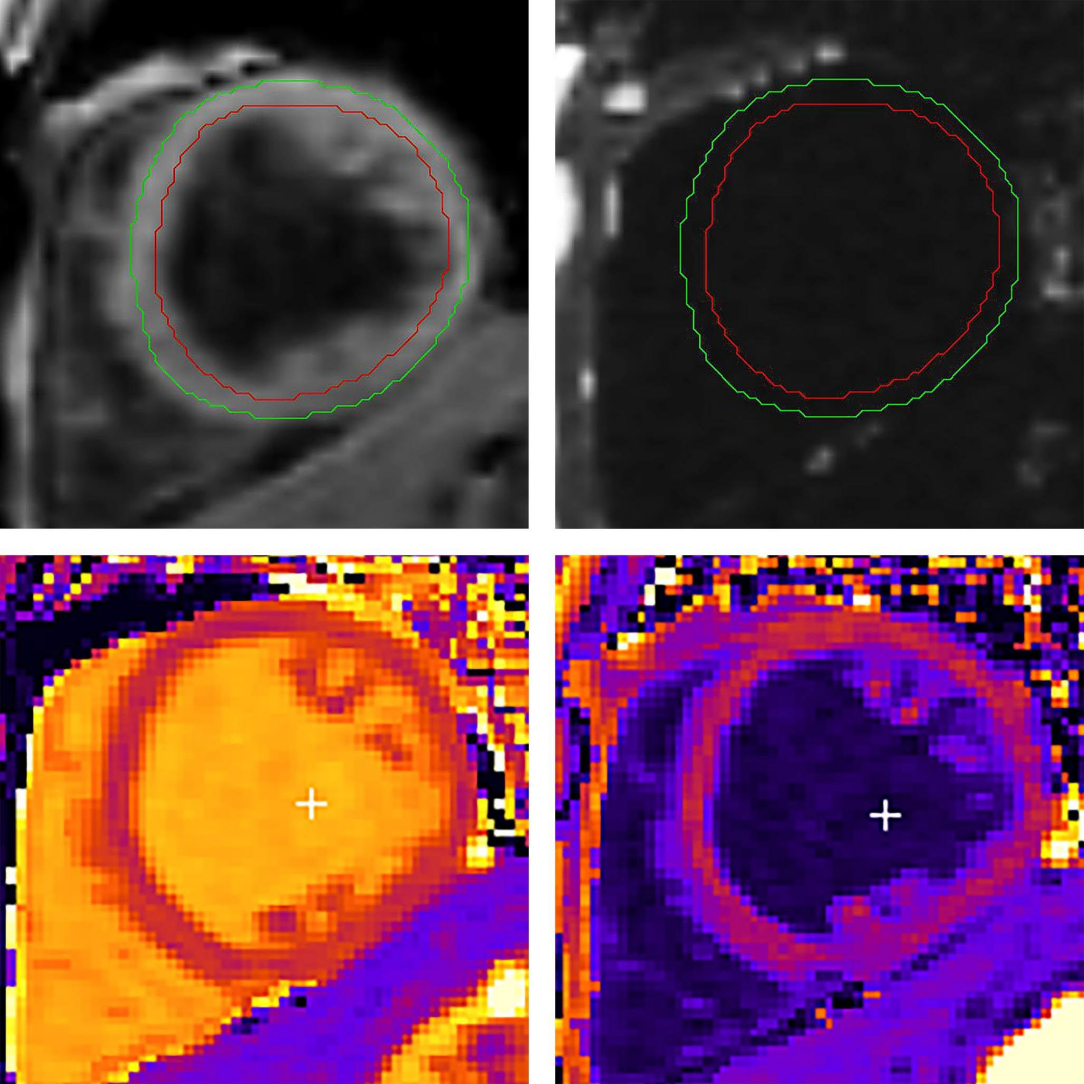
Quantification of LV-myoFat fraction by VARPRO (top panels) and ECV-fraction by T1 mapping (bottom panels). Epicardial and endocardial contours are drawn in the mid-myocardium to avoid contamination by the epicardial adipose tissue and LV blood pool. Top left and right panels show the water-only and fat-only images respectively. LV-myoFat fraction is calculated as the ratio of mean pixel signal intensity of LV fat-only image to mean pixel signal intensity of LV water-only image, and expressed as a percentage. Bottom left and right panels show the native T1 and post-contrast T1 maps respectively. ECV-fraction is calculated as per previous publication [8]. ECV: extracellular volume, LV-myoFat: left ventricular myocardial fat content, LV: left ventricular, VARPRO: water and fat separated imaging with variable projection

The LV basal and mid-ventricular short-axis slices by the VARPRO sequence were obtained at identical LV levels as quantification of interstitial fibrosis by ECV. During analysis, epicardial and endocardial contours were deliberately drawn in the mid-myocardium to avoid partial volume effects from epicardial adipose tissue and LV blood pool (Fig.1) [2]. LV-myoFat fraction was calculated and expressed as a percentage:

$$LV\text{-}myoFat\ fraction= \frac{mean\ pixel\ signal\ intensity\ of\ LV\ fat\ only\ image}{mean\ pixel\ signal\ intensity\ or\ LV\ water\ only\ image}\times 100\%$$

This equation is identical to the quantification of intramyocardial triglyceride (TG) content by spectroscopy [2]. Total LV-myoFat volume was calculated as:

$$Total\ LV\text{-}myoFat\ volume = LV\text{-}myoFat\ fraction \times myocardial\ fat\ volume$$;

$$myocardial\ fat\ volume \left(mL\right)= \frac{LV\ mass \left(g\right)}{density\ of\ fat (i.e., 0.9g/mL)}$$

#### MRI quantification of LV interstitial fibrosis by ECV

The MOLLI T1 mapping sequence was used to quantify the burden of interstitial fibrosis by ECV [8]. See Supplemental Appendix for ECV imaging parameters.

was calculated and expressed as a percentage [8]:

$$ECV\text{-}fraction=\left(1-hematocrit\right)\times \frac{\left(\frac{1}{post\ contrast\ myocardial T1}-\frac{1}{native\ myocardial\ T1}\right)}{\left(\frac{1}{post\ contrast\ blood T1}-\frac{1}{native\ blood\ T1}\right)}$$

Total myocardial interstitial volume was calculated as previously published [12]:

$$Total\ myocardial\ interstitial\ volume=ECV\text{-}fraction \times myocardial\ muscle\ volume$$;

$$myocardial\ muscle\ volume \left(mL\right)= \frac{LV\ mass \left(g\right)}{density\ of\ myocardium (i.e., 1.05g/mL)}$$

### Echocardiography

Transthoracic echocardiography was performed using commercially available system and images were analyzed offline (Vivid E9, EchoPAC version 113, GE-Vingmed, Horten, Norway). See Supplementary Appendix for the Doppler measurements.

LV-GLS were derived from 2-dimensional images in the apical 2-, 3- and 4-chamber views, at 50–70 frame/s. During analysis, the endocardial border was manually traced at end-systole and the region of interest width adjusted to include the entire myocardium. Our group has previously reported the intra- and interobserver measurement variability for LV-GLS in patients with increased BMI [13].

### Statistical analysis

See Supplemental Appendix for sample size calculation. All continuous variables were tested for Gaussian distribution. Continuous variables were presented as mean±1 SD unless, and categorical variables were presented as percentages. Chi-square and Fisher’s exact test were used as appropriate to compare categorical variables between groups. One-way analysis of variance (ANOVA) was used to compare the 3 groups of patients for continuous variables, and pairwise comparisons (by unpaired Student’s t-test and Mann-Whitney U test as appropriate) for significant results by ANOVA were performed with Bonferroni corrections. Pearson correlation was used to determine the association between 2 continuous variables. Multiple linear regression analyses were performed to identify independent variables associated with LV-myoFat fraction, total LV-myoFat volume, ECV-fraction, total myocardial interstitial volume, and LV-GLS using the backward elimination method. In each multivariable model, significant univariables with p < 0.05 were entered as covariates. In addition, age was forced into multivariable analyses due to significant differences between the 3 groups of patients. A tolerance of > 0.5 was set to avoid multicollinearity. RPP was used in the multivariable models instead of heart rate and BP due to its higher correlation coefficient and to prevent overfitting of the multivariable models. A 2-tailed p value < 0.05 was considered significant. Statistical analyses were performed using IBM SPSS Statistics for Windows, version 21.0 (Armonk, NY).

## Results

Table1 outlines all the baseline characteristics. There were significant differences in age, HbA1c, HOMA-IR, fasting lipid profile, heart rate, systolic BP and RPP between the 3 groups of patients. However, there was no difference in BMI between Group 2 vs. Group 3.

**Table 1:** Clinical, echocardiographic and MRI characteristics

Variable	Total population (n = 100)	Group 1 (n = 27)	Group 2 (n = 21)	Group 3 (n = 52)	p value*
**Clinical**					
Age (year)	46 ± 14	30 ± 4	45 ± 13^†^	55 ± 9^†§^	< 0.001
Male gender	60.0%	55.6%	66.7%	59.6%	0.74
BMI (kg/m^2^)	29.2 ± 6.6	22.0 ± 1.8	30.3 ± 6.1^†^	32.6 ± 5.4^†^	< 0.001
Waist/hip ratio	0.94 ± 0.11	0.82 ± 0.07	0.94 ± 0.13^†^	1.01 ± 0.07^†^	< 0.001
Active smoking	10%	0%	0%	19.2%^†§^	0.006
Hypertension	35%	0%	0%	67.3%^†§^	< 0.001
Heart rate (beats/min)	66 ± 12	63 ± 10	59 ± 9	70 ± 13^§^	0.001
Systolic blood pressure (mmHg)	135 ± 18	127 ± 9	131 ± 16	142 ± 20^†^	< 0.001
Diastolic blood pressure (mmHg)	81 ± 10	78 ± 9	81 ± 9	82 ± 11	0.19
RPP (beats/min.mmHg)	8860 ± 1979	7958 ± 1395	7733 ± 1362	9866 ± 2045^†§^	< 0.001
**Medications**					
Antiplatelet	22.0%	0%	0%	42.3%^†§^	< 0.001
Beta blockers	18.0%	0%	0%	34.6%^†§^	< 0.001
Calcium channel blockers	10.0%	0%	0%	19.2%^†§^	0.006
ACEI/ARB	31.0%	0%	0%	59.6%^†§^	< 0.001
HMG-CoA reductase inhibitor	33.0%	0%	0%	63.5%^†§^	< 0.001
Metformin	42.0%	0%	0%	80.8%^†§^	< 0.001
Sulphonylurea	13.0%	0%	0%	25.0%^†§^	0.001
DPP4 inhibitors	9.0%	0%	0%	17.3%^†§^	0.010
GLP1 receptor agonist	2.0%	0%	0%	3.8%	0.39
Insulin	15.0%	0%	0%	28.8%^†§^	< 0.001
**Biochemical**					
Total cholesterol (mmol/L)	4.7 ± 1.1	4.7 ± 0.7	5.0 ± 0.6	4.5 ± 1.3	0.15
HDL cholesterol (mmol/L)	1.3 ± 0.4	1.6 ± 0.4	1.3 ± 0.3	1.1 ± 0.2^†§^	< 0.001
LDL cholesterol (mmol/L)	2.5 ± 0.9	2.7 ± 0.5	3.2 ± 0.6^†^	2.0 ± 0.9^†§^	< 0.001
Plasma TG (mmol/L)	2.4 ± 2.6	0.9 ± 0.5	1.2 ± 0.4	3.6 ± 3.1^†§^	< 0.001
Plasma glucose (mmol/L)	8.0 ± 4.1	4.9 ± 0.4	5.2 ± 0.5	10.7 ± 4.0^†§^	< 0.001
Hemoglobin (g/L)	142 ± 14	143 ± 14	148 ± 14	139 ± 13	0.07
HbA1c (%)	6.8 ± 1.9	5.2 ± 0.3	5.3 ± 0.2	8.2 ± 1.6^†§^	< 0.001
HOMA-IR	7.7 ± 10.7	0.9 ± 0.8	1.7 ± 1.2^†^	10.4 ± 11.7^†§^	< 0.001
High-sensitivity CRP (mg/L)	2.8 ± 3.6	1.2 ± 1.9	1.4 ± 1.1	3.7 ± 4.1^†§^	0.007
**Cardiac magnetic resonance imaging**					
LV mass (g)	95 ± 28	78 ± 17	87 ± 21	99 ± 29	0.001
LVEDV (mL)	162 ± 34	167 ± 35	173 ± 31	161 ± 36	0.40
LVESV (mL)	75 ± 21	74 ± 16	79 ± 18	75 ± 22	0.68
LVEF (%)	54 ± 5	55 ± 3	55 ± 5	54 ± 6	0.52
LV-myoFat fraction (%)	7.3 ± 2.7	4.1 ± 0.8	5.3 ± 0.9^†^	8.4 ± 2.3^†§^	< 0.001
Total LV-myoFat volume (mL)	7.9 ± 4.4	3.1 ± 1.1	5.3 ± 1.6^†^	9.6 ± 4.3^†§^	< 0.001
ECV-fraction (%)	28.7 ± 2.6	27.0 ± 0.3	27.9 ± 1.1	29.2 ± 3.0^†§^	0.03
Total interstitial volume (mL)	25.7 ± 8.0	18.6 ± 5.3	23.1 ± 5.5	27.8 ± 8.2^†^	0.002
**Echocardiography**					
Transmitral E/A ratio	1.36 ± 0.68	1.95 ± 0.77	1.53 ± 0.68	0.97 ± 0.26^†§^	< 0.001
Deceleration time (ms)	184 ± 46	167 ± 28	173 ± 41	196 ± 52^†^	0.01
Septal E’ velocity (cm/s)	9.0 ± 3.7	13.4 ± 1.9	10.0 ± 2.8^†^	6.2 ± 1.5^†§^	< 0.001
Septal E/e’	9.1 ± 3.6	5.9 ± 1.2	7.9 ± 2.1^†^	11.3 ± 3.3^†§^	< 0.001
LV-GLS (%)	-17.2 ± 2.6	-19.6 ± 1.8	-18.0 ± 2.0^†^	-15.7 ± 2.1^†§^	< 0.001

*p value by one-way ANOVA; ^†^p < 0.05 vs. Group 1 with Bonferroni correction; ^§^p < 0.05 vs. Group 2 with Bonferroni correction. ACEI/ARB: angiotensin converting enzyme inhibitor/angiotensin receptor blockers; BMI: body mass index; CRP: c-reactive protein; DPP4: dipeptidyl peptidase 4; ECV: extracellular volume; EDV: end-diastolic volume; ESV: end-systolic volume; EF: ejection fraction; GLP1: glucagon-like peptide 1; GLS: global longitudinal strain; HbA1c: glycated hemoglobin; HDL: high density lipoprotein; HOMA-IR: homeostatic model assessment index of insulin resistance; LDL: low density lipoprotein; LV: left ventricular; LV-myoFat: left ventricular myocardial fat content; RPP: rate pressure product; TG: triglyceride.

### Correlates of LV-myoFat fraction

**Fig. 2:**
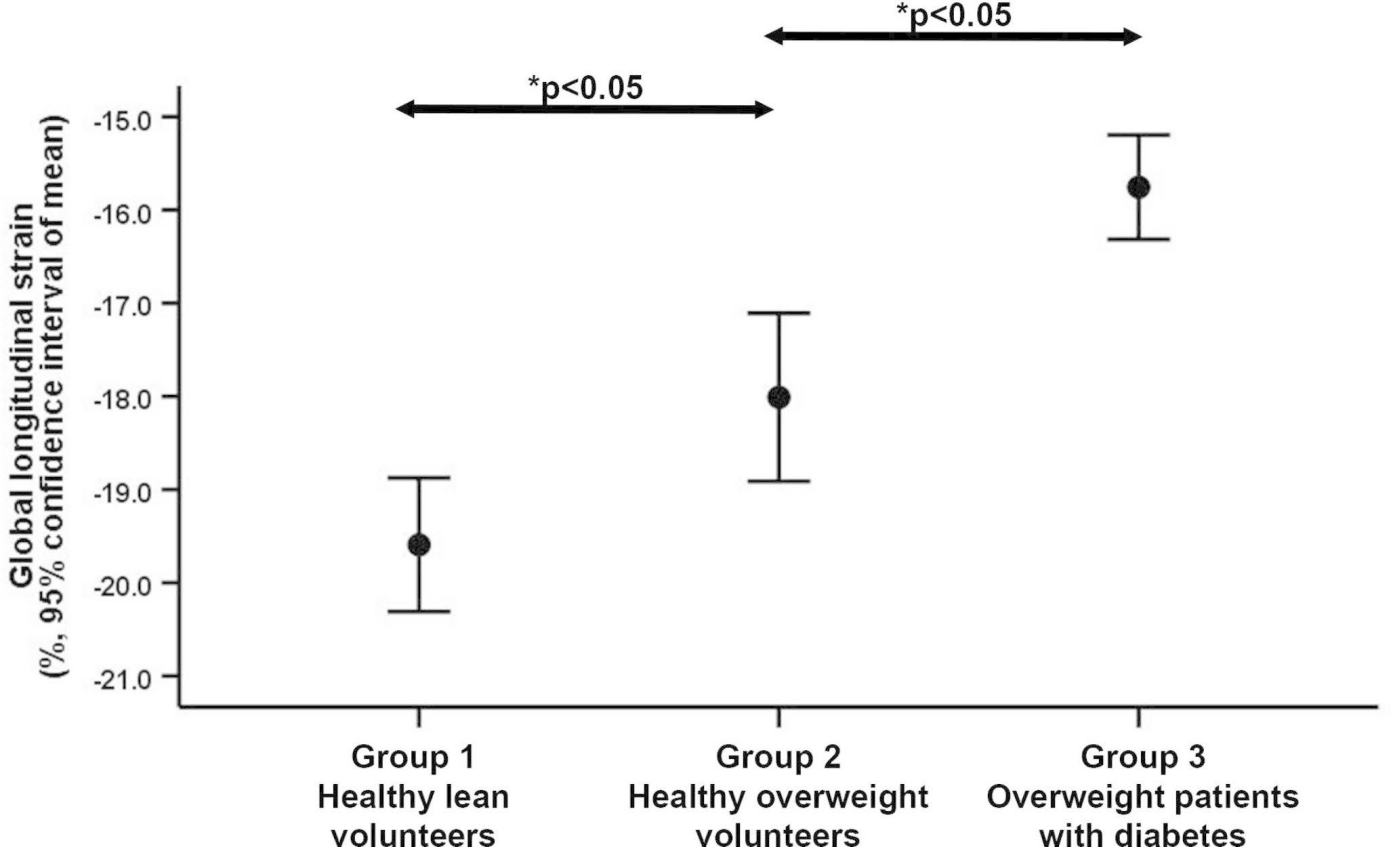
Error bars showing progressive increase in LV-myoFat fraction in the 3 groups. LV-myoFat: left ventricular myocardial fat content. *Multiple comparisons performed with Bonferroni correction

There was no difference in LV-myoFat fraction between men vs. women (6.6 ± 2.4 vs. 6.6 ± 3.1%, p > 0.99), but there were significant differences in LV-myoFat fraction between the 3 groups (all p < 0.05 with Bonferroni correction; Fig.2). Table2 shows that LV-myoFat fraction increased with older age, increasing BMI, HOMA-IR, HbA1c, plasma TG, and lower LDL- and HDL-cholesterol. LV-myoFat fraction was also correlated with heart rate (r = 0.37, p < 0.001), systolic BP (r = 0.20, p = 0.05) and RPP (r = 0.41, p < 0.001). Only diabetes was included in the multivariable model due to significant collinearity with HbA1c and better correlation with LV-myoFat fraction. On multivariable analysis, BMI, presence of diabetes and HOMA-IR were independently associated with LV-myoFat fraction (model R = 0.82; Table2, model 1). The results did not change when RPP was changed into heart rate and systolic BP. Age was not an independent determinant of LV-myoFat fraction for both multivariable models.

**Table 2:** Significant univariable and multivariable determinants of left ventricular myocardial fat fraction

	Univariable		Multivariable Model 1		Multivariable Model 2	
Variable	Standardized β	p value	Standardized β	p value	Standardized β	p value
BMI	0.662	< 0.001	0.299	< 0.001		
Presence of diabetes	0.728	< 0.001	0.449	< 0.001		
Presence of diabetes* BMI interaction	0.807	< 0.001			0.676	< 0.001
Age	0.476	< 0.001				
RPP	0.412	< 0.001				
HbA1c	0.652	< 0.001				
HOMA-IR	0.610	< 0.001	0.250	0.001	0.218	0.003
Plasma TG	0.374	< 0.001				
LV mass	0.392	< 0.001				

BMI: body mass index; HbA1c: glycated hemoglobin; HOMA-IR: homeostatic model assessment index of insulin resistance; LV: left ventricular; RPP: rate pressure product; TG: triglyceride.

Variables included in multivariable model 1: BMI, presence of diabetes, age, RPP, HOMA-IR, plasma TG, LV mass

Variables included in multivariable model 2: model 1 + presence of diabetes*BMI interaction

Next, the presence of diabetes*BMI interaction term was included in the multivariable model to determine if diabetic patients had higher LV-myoFat fraction with increasing BMI compared to non-diabetic patients (Table2, model 2). The multivariable model showed that the presence of diabetes*BMI interaction term and HOMA-IR were independently associated with LV-myoFat fraction (model R = 0.82). The presence of diabetes*BMI interaction term was the strongest determinant of LV-myoFat fraction.

When BMI was replaced by waist/hip ratio, the multivariable results did not change and the presence of diabetes*waist/hip ratio interaction term (standardized β = 0.556, p < 0.001) and HOMA-IR (standardized β = 0.345, p < 0.001) remained independent determinants of LV-myoFat fraction (model R = 0.78). The supplemental appendix showed the determinants of total LV-myoFat volume.

### Correlates of ECV-fraction

**Fig. 3:**
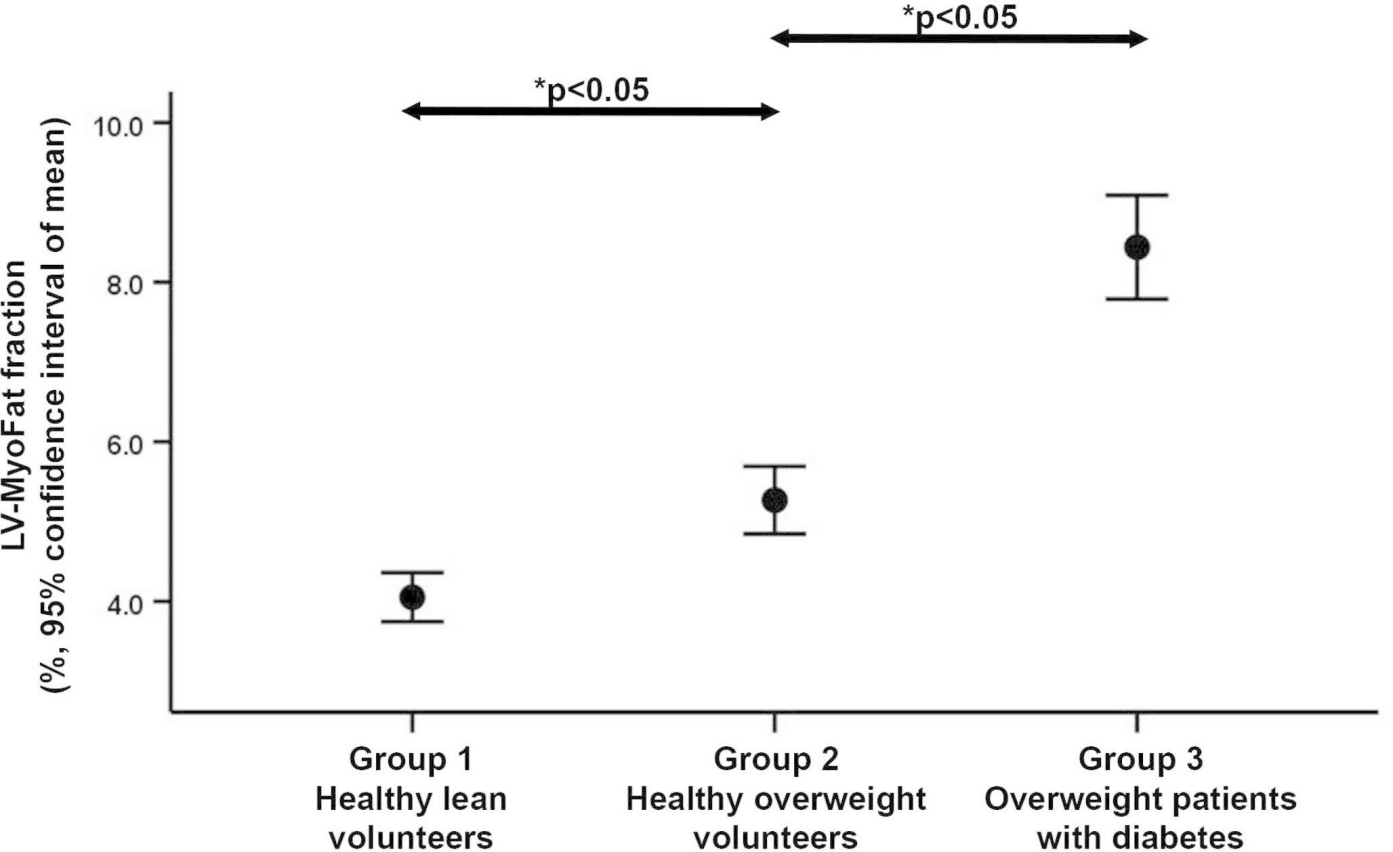
Error bars showing ECV-fraction in the 3 groups of subjects. Group 3 had significantly higher ECV compared to Groups 1 and 2. ECV: extracellular volume, LV: left ventricular. *Multiple comparisons performed with Bonferroni correction

There was no difference in ECV-fraction between men vs. women (28.3 ± 2.4 vs. 29.2 ± 2.8%, p = 0.12). Table1 showed that there was no difference in ECV-fraction between Group 1 and Group 2, but Group 3 had significantly higher ECV-fraction compared to the other 2 groups (both p < 0.05 with Bonferroni correction; Fig.3). Table3 shows that ECV-fraction was significantly correlated with BMI (r = 0.300, p = 0.009), heart rate (r = 0.260, p = 0.028), HbA1c (r = 0.430, p < 0.001) and LV-myoFat fraction (r = 0.446, p < 0.001). In contrast, ECV-fraction was not correlated with age, hypertension, systolic BP, RPP or HOMA-IR. To identify independent correlates of ECV-fraction, significant univariables (BMI, diabetes, heart rate, plasma TG, and LV-myoFat fraction) were entered as covariates into the multiple linear regression model. As before, HbA1c was not included in the ECV-fraction regression model due to significant collinearity with diabetes. On multivariable analysis, only LV-myoFat fraction was independently associated with ECV-fraction (model R = 0.45).

Next, age and hypertension were forced into the multivariable model to determine if they were associated with ECV-fraction. However, the results did not change and LV-myoFat fraction remained the only significant independent determinant. Similarly, to determine if diabetic patients had higher ECV-fraction with increasing BMI compared to non-diabetic patients, the presence of diabetes*BMI interaction term was also included in the multivariable model together with age and hypertension. However, the results did not change and LV-myoFat fraction remained the only significant independent determinant of ECV-fraction.

**Table 3:** Significant univariable and multivariable determinants of left ventricular extracellular volume fraction

	**Univariable**		**Multivariable**	
Variable	Standardized β	p value	Standardized β	p value
BMI	0.300	0.009		
Presence of diabetes	0.293	0.011		
Age	0.067	0.57		
Heart rate	0.260	0.028		
Plasma TG	0.228	0.049		
LV-myoFat fraction	0.446	< 0.001	0.446	< 0.001

BMI: body mass index; HbA1c: glycated hemoglobin; LV: left ventricular; LV-myoFat: left ventricular myocardial fat content; TG: triglyceride

The supplemental appendix showed the determinants of total myocardial interstitial volume.

### Correlates of LV-GLS

**Fig. 4:**
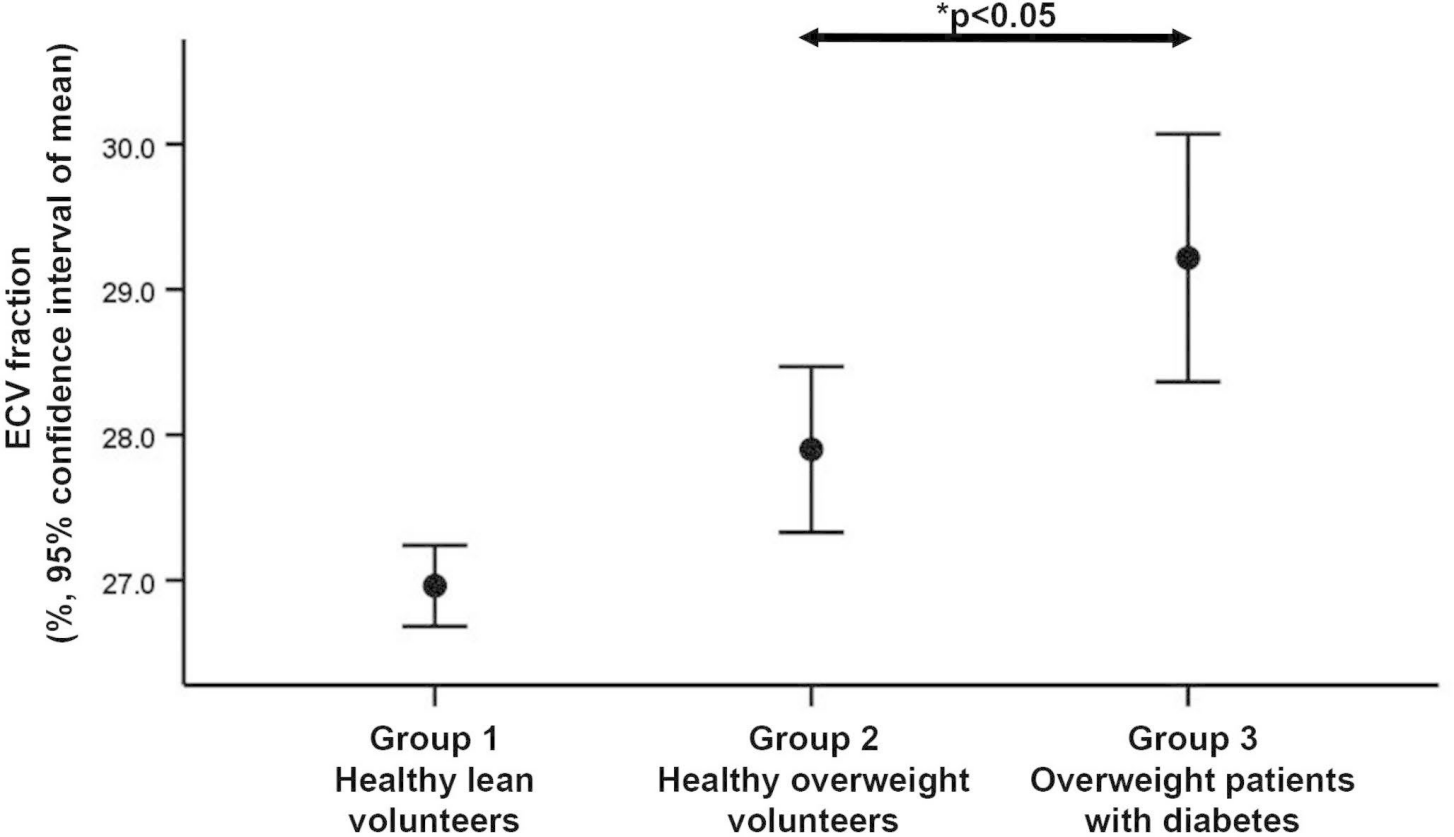
Error bars showing progressively more impaired LV-GLS in the 3 groups. GLS: global longitudinal strain, LV: left ventricular. *Multiple comparisons performed with Bonferroni correction

There was a trend towards lower LV-GLS in men than women (-16.8 ± 2.2 vs. -17.9 ± 3.1%, p = 0.056). Figure4 shows a progressive decline in LV-GLS in the 3 groups (p < 0.001 by ANOVA). Table4 shows all the significant univariable correlations with LV-GLS. On multivariable analysis, the presence of diabetes*BMI interaction term, RPP and LV mass were independently associated with LV-GLS (model R = 0.75; Table4, model 1). When RPP was replaced by heart rate and systolic BP, the presence of diabetes*BMI interaction term, heart rate and LV mass were independently associated with LV-GLS (model R = 0.75). Age was not an independent determinant of LV-GLS on both multivariable models.

**Table 4:** Significant univariable and multivariable determinants of left ventricular global longitudinal strain

	**Univariable**		**Multivariable Model 1**		**Multivariable Model 2**		**Multivariable Model 3**		
Variable	Standardized β	p value	Standardized β	p value	Standardized β	p value	Standardized β	p value	
BMI	0.545	< 0.001							
Presence of diabetes	0.623	< 0.001							
Presence of diabetes* BMI interaction	0.692	< 0.001	0.451	< 0.001					
Age	0.437	< 0.001							
Heart rate	0.359	< 0.001							
Systolic blood pressure	0.302	0.002							
RPP	0.467	< 0.001	0.214	0.011	0.298	0.002	0.283	0.002	
HOMA-IR	0.512	< 0.001							
Plasma TG	0.351	< 0.001							
LV mass	0.510	< 0.001	0.284	< 0.001	0.350	< 0.001	0.327	< 0.001	
LV-myoFat fraction	0.646	< 0.001			0.248	0.028			
ECV-fraction	0.324	0.005			0.201	0.043			
LV-myoFat fraction* ECV-fraction interaction	0.560	< 0.001					0.382	< 0.001	

BMI: body mass index; ECV: extracellular volume; LV: left ventricular; LV-myoFat: left ventricular myocardial fat content; RPP: rate pressure product; TG: triglyceride

Variables included in multivariable model 1: BMI, presence of diabetes, presence of diabetes*BMI interaction, age, RPP, plasma TG, LV mass, HOMA-IR

Variables included in multivariable model 2: age, RPP, plasma TG, LV mass, LV-myoFat fraction, ECV-fraction

Variables included in multivariable model 3: model 2 + LV-myoFat fraction*ECV-fraction interaction

To further explore the role of LV myocardial steatosis and interstitial fibrosis on LV-GLS, the presence of diabetes and BMI variables were replaced with LV-myoFat fraction and ECV-fraction (Table4, model 2). Presence of diabetes and BMI were not included together with LV-myoFat fraction and ECV-fraction due to significant multicollinearity. On multivariable analysis, LV-myoFat fraction, ECV-fraction, RPP and LV mass were independently associated with LV-GLS (model R = 0.71). Age was not an independent determinant of LV-GLS in this multivariable model.

Next, LV-myoFat fraction*ECV-fraction interaction term was added to the multivariable model together with LV-myoFat fraction, ECV-fraction, age, RPP, plasma TG and LV mass (Table4, model 3). On multivariable analysis, LV-myoFat fraction*ECV-fraction interaction term, RPP and LV mass were independently associated with LV-GLS (model R = 0.70). When RPP was replaced with heart rate and systolic BP, the independent determinants of LV-GLS were LV-myoFat fraction*ECV-fraction interaction term, heart rate, and LV mass (model R = 0.70). Age was not an independent determinant of LV-GLS in this multivariable analysis.

Supplementary Table3 shows that when LV-myoFat fraction and ECV-fraction were replaced by total LV-myoFat volume and total myocardial interstitial volume respectively, the only independent determinants of LV-GLS were total LV-myoFat volume*total myocardial interstitial volume interaction term and RPP (model R = 0.64).

## Discussion

The present study showed that presence of diabetes, BMI and insulin resistance were independently associated with increased myocardial steatosis. Furthermore, there was an interaction between diabetes and BMI, indicating that diabetic patients had a greater degree of steatosis with increasing BMI compared to non-diabetic patients. In turn, increased myocardial steatosis was independently associated with a higher burden of interstitial fibrosis. Finally, diabetic patients had more impaired LV-GLS with increasing BMI compared to non-diabetic patients, and both the extent of myocardial steatosis and burden of interstitial fibrosis were independently associated with LV-GLS.

### Myocardial steatosis

Quantifying myocardial TG by the gold standard proton magnetic resonance spectroscopy is challenging and time consuming due to the need to perform double respiratory and cardiac gating [14]. In contrast, VARPRO has the advantages of easy quantification of regional myocardial fat content beyond the interventricular septum, avoidance of partial volume effects from contamination by epicardial adipose tissue, faster image acquisition using a single breath-hold, and most importantly the ability to couple with multiparametric imaging such as T1 mapping to ensure identical slice positioning.

Previous validation study showed that VARPRO overestimates myocardial TG concentration due to its T1 bias from the high flip angle [15]. However, lower flip angles will reduce the signal-to-noise ratio leading to incorrect fat fraction estimation, and can prevent accurate myocardial contouring due to increased image noise [16]. We have previously demonstrated that the overestimation by VARPRO is linear, and it still had excellent correlation and consistency against spectroscopy [2]. Therefore, VARPRO could be used as a surrogate when quantifying myocardial TG.

Both the associations between diabetes and myocardial steatosis, and increased BMI without diabetes and myocardial steatosis, have previously been well documented [17, 18]. However, no studies to date have evaluated the combined effects of diabetes and increased BMI on myocardial TG content. In contrast, the present study’s design permitted the combined evaluation of both increased BMI and diabetes on myocardial steatosis. The interaction between diabetes and BMI on myocardial TG content suggested that diabetic patients had a greater degree of steatosis with increasing BMI compared to non-diabetic patients. Although age was correlated with increased myocardial steatosis on univariable analyses, it was not an independent determinant on multivariable analyses after including metabolic variables such as BMI, insulin resistance, and diabetes.

### Myocardial interstitial fibrosis

This study showed that increasing BMI was correlated with a higher burden of myocardial interstitial fibrosis on univariable analyses, and diabetic patients had significantly more myocardial interstitial fibrosis compared to non-diabetic patients. However, post-hoc subgroup analysis corrected for multiple comparisons suggested no significant difference in myocardial interstitial fibrosis between normal weight vs. increased BMI in non-diabetic healthy volunteers. On multivariable analysis, myocardial steatosis was independently associated with myocardial interstitial fibrosis. As the healthy volunteers with increased BMI in Group 2 had no underlying cardiac comorbidities could have potentially confounded the LV-myoFat and ECV measurements, the results suggested that isolated increased BMI without other cardiac comorbidities was not associated with increased myocardial interstitial fibrosis.

Increasing age is often assumed to be associated with increased interstitial fibrosis and myocardial dysfunction. However, age was not independently associated with ECV-fraction or total myocardial interstitial volume in the present study. This was consistent with a previous MRI study that similarly showed no correlation between age and ECV in healthy volunteers [19].

Previous study showed that increased fat content can bias native T1 mapping [20]. Specifically, native T1 time can increase with out-of-phase inversion recovery imaging protocols, and decrease with in-phase imaging protocols. In contrast, because ECV is a ratio, increased myocardial fat content is unlikely to bias it “true” value as stated by current expert consensus statement [21].

### Myocardial function

Patients with obesity and diabetes are more likely to develop HFpEF [22, 23]. In the Framingham study, obese patients had double the risk of heart failure compared to patients with normal BMI [24]. However, few studies to date have evaluated the combined effects of increased BMI and diabetes on LV-GLS [7, 25]. Both obesity and diabetes can individually cause myocardial dysfunction independent of coronary artery disease and hypertension due to shared myocardial pathophysiological abnormalities such as altered myocardial energetics, abnormal substrate usage with steatosis, cardiac autonomic neuropathy, increased interstitial collagen deposition [26]. These processes initially causes subclinical myocardial dysfunction, but there is eventual overt development of clinical HFpEF. It has been shown that a significant proportion of patients with HFpEF have impaired LV-GLS [27].

We recently showed that both diabetes and increased BMI had an additive detrimental effect on LV-GLS [7]. The present study expanded our previous findings by exploring the underlying combined effects of myocardial steatosis and interstitial fibrosis on LV-GLS. Between the 3 groups of patients, there were incremental increases in LV-myoFat fraction (Fig.1) and corresponding declines in LV-GLS (Fig.3). However, ECV-fraction was only increased in the presence of diabetes (Fig.2). On multivariable analysis, the presence of diabetes*BMI interaction term was independently associated with LV-GLS, indicating that diabetic patients had more impaired LV-GLS with increasing BMI compared to non-diabetic patients. When these clinical variables (i.e., presence of diabetes and BMI) were replaced with imaging parameters (i.e., LV-myoFat fraction and ECV-fraction), both variables were independently associated with LV-GLS. Furthermore, there was an interaction between LV-myoFat fraction and ECV-fraction on LV-GLS, suggesting that as BMI increases in non-diabetic patients, the decline in LV-GLS was partly secondary to increased myocardial steatosis without a corresponding increase in interstitial fibrosis. However, once patients develop type 2 diabetes, the additional decline in LV-GLS was partly due to increased myocardial interstitial fibrosis.

The present study did not show an independent relationship between age and LV-GLS. This was consistent with other publications including the Framingham study that failed to show any age-related changes in LV-GLS [28, 29]. In contrast, any age-related decline in LV-GLS in the elderly are likely secondary to subclinical myocardial disease [30]. Importantly, current guidelines’ statement of a possible age-related decline in LV-GLS was actually based on a single study of 480 subjects (37.9% with hypertension) using tissue Doppler derived longitudinal strain [31, 32].

### Study limitations

Although we have previously validated VARPRO against spectroscopy in normal healthy volunteers of varying BMI, it has not been validated in diabetic patients [2].

### Clinical implications

It is well accepted that LV-GLS is superior to LVEF in detecting subclinical myocardial dysfunction, and is prognostic for long term adverse clinical outcomes [7, 33]. The present study suggest that measures aimed at reducing myocardial steatosis in non-diabetic patients with HFpEF (e.g. weight loss) may lead to improvement in LV-GLS. In contrast, therapies aiming to reduce myocardial interstitial fibrosis may be more pertinent for diabetic patients with HFpEF and impaired LV-GLS.

## Conclusion

The present study demonstrated that diabetes, increased BMI, and insulin resistance were independently associated with increased myocardial steatosis. In turn, the extent of myocardial steatosis was independently associated with the burden of myocardial interstitial fibrosis. Finally, both increased BMI and diabetes were independently and incrementally associated with more impaired LV-GLS. Multivariable analysis suggested that LV-GLS was determined by myocardial steatosis and interstitial fibrosis. The changes in myocardial steatosis, interstitial fibrosis and LV-GLS were independent of age.

### Clinical perspectives

#### Competencies in medical knowledge

We demonstrated that both increased BMI and diabetes are independently associated with increased myocardial steatosis, which in turn is related with a greater burden of interstitial fibrosis. Together, both increased myocardial steatosis and interstitial fibrosis is correlated with impaired LV-GLS. This study provides insight into the mechanism in which patients with increased body weight and patients with diabetes can develop heart failure with preserved ejection fraction.

### Translational outlook

Tissue characterization by cardiac MRI can provide novel insights into the pathophysiological mechanisms that predispose patients with increased body weight and patients with diabetes to develop heart failure with preserved ejection fraction. More studies are needed to determine how weight loss and improved glycemic control can alter myocardial composition and function.

## Electronic supplementary material

Below is the link to the electronic supplementary material.

Supplementary Material 1

Supplementary Material 2

## Abbreviation List

2D: 2-dimensional
BMI: body mass index
BP: blood pressure
ECV: extracellular volume
EDV: end-diastolic volume
EF: ejection fraction
ESV: end-systolic volume
FOV: field of view
GLS: global longitudinal strain
HbA1c: glycated hemoglobin
HDL: high density lipoprotein
HFpEF: heart failure with preserved ejection fraction
HOMA-IR: homeostatic model assessment index of insulin resistance
LDL: low density lipoprotein
LV: left ventricle / ventricular
LV-myoFat: myocardial fat
MRI: magnetic resonance imaging
RPP: rate pressure product
SFFP: balanced steady state free-precession
TE: echo time
TG: triglyceride
TR: repetition time
VARPRO: water and fat separated imaging with variable projection
