# Supplementary material for: Impact of body mass index and diabetes on myocardial fat content, interstitial fibrosis and function
Source: Int J Cardiovasc Imaging. 2022 Oct 28;39(2):379–90. doi: 10.1007/s10554-022-02723-8 (PMC9870836; doi:10.1007/s10554-022-02723-8)
Supplement: Supplementary file 1 — Supplementary Material 1 [file 10554_2022_2723_MOESM1_ESM.docx]

# Acknowledgements

The authors acknowledge Dr Lisa Gillinder and Dr Shi Yi Goo who contributed in the acquisition and collection of some of the data.

# Conflicts of Interest

The Department of Cardiology of Leiden University Medical Centre received grants from Abbott Vascular, Bayer, Biotronik, Bioventrix, Medtronic, Boston Scientific Corporation, Edwards Lifesciences and GE Healthcare. The study was supported by an unrestricted educational grant by Abbott Australasia Pty Ltd. Jeroen Bax received speaker fees from Abbott Vascular and Medtronic. Victoria Delgado received speaker fees from Abbott Vascular, Edwards Lifesciences, GE healthcare, and Medtronic. The remaining authors have no conflicts of interest to disclose.

# Sources of funding

We gratefully acknowledge the assistance of Siemens Healthcare for the provision of the VARPRO sequences.
