# Supplementary material for: Impact of body mass index and diabetes on myocardial fat content, interstitial fibrosis and function
Source: Int J Cardiovasc Imaging. 2022 Oct 28;39(2):379–90. doi: 10.1007/s10554-022-02723-8 (PMC9870836; doi:10.1007/s10554-022-02723-8)
Supplement: Supplementary file 2 — Supplementary Material 2 [file 10554_2022_2723_MOESM2_ESM.docx]

**SUPPLEMENTAL METHODS**

Hematocrit was measured using the cumulative pulse height method (Sysmex analyzer, Sysmex America, Lincolnshire, IL, USA).(1) High-density lipoprotein (HDL) cholesterol was measured as a homogeneous assay in liquid phase (Hitachi 747 autoanalyzer, Boehringer Mannheim, Mannheim, Germany). Low-density lipoprotein (LDL) cholesterol was calculated using the Friedewald equation.(2) If fasting plasma triglyceride (TG) is ≥4.5mmol/L, LDL cholesterol was directly measured by ultracentrifugation and electrophoresis. Fasting plasma glucose was measured by enzymatic assay (Dade Behring, Newark, DE, USA). Serum insulin was evaluated using a chemiluminescent enzyme immunoassay (Immulite 2000; Diagnostic Products, Los Angeles, CA, USA). HbA1c measurements were performed using high performance liquid chromatography cation-exchange analyzers (Bio-Rad D-10^TM^, Bio-Rad Laboratories, Hercules, CA, USA).

## Cardiac magnetic resonance imaging

Typical imaging parameters for cine images were: balanced steady state free-precession (SFFP), echo time (TE)=1.0ms, repetition time (TR)=58ms, flip angle=54°, slice thickness=8mm, slice gap=2mm, field of view (FOV)=340x340mm, reconstructed matrix size=156x192.

*MRI quantification of myocardial fat content*

The VARPRO sequence was ECG triggered, 2 R-R intervals between inversions, and used an echo-train readout with 4 echoes. Typical imaging parameters were: gradient echo, TE=1.27, 3.18, 5.09, 7.00ms, TR=161ms, flip angle=24°, slice thickness=6mm, FOV=360x292mm, reconstructed matrix size=256x218, bandwidth=1502Hz/pixel.

*MRI quantification of LV interstitial fibrosis by ECV*

All subjects received 0.1mmol/kg of gadolinium diethylenetriamine penta-acetic acid. Typical imaging parameters were: single-shot SFFP, TE=1.0ms, TR=2.7ms, flip angle=35°, slice thickness=8mm, FOV=340x340mm, reconstructed matrix size=106x192 pixels, trigger delay=300ms.

## Echocardiography

Transmitral E and A velocities, and deceleration time, were recorded using pulsed-wave Doppler in the apical 4-chamber view using a 2mm sample volume placed at the mitral leaflet tips. Mitral annular early diastolic (e’) velocity was recorded using pulsed-wave tissue Doppler placed at the septal mitral annulus at end-expiration. Transmitral E wave to mitral annular e’ velocity (E/e’) ratio was calculated.

## Statistical analysis

Statistical power analysis software G*Power (University of Düsseldorf, Düsseldorf, Germany) was used to calculate effective sample size to detect a difference in LV-GLS in the 3 groups.(3) We assumed most healthy volunteers in Group 2 would be overweight (i.e. BMI 25.0–29.9kg/m^2^) instead of obese (BMI ≥30kg/m^2^), resulting in a smaller absolute difference in LV-GLS compared to Group 1. Furthermore, we used an unequal cell sample size ratio of 1:1:2 for Group 1, Group 2, and Group 3 respectively because we assumed greater difficulty in recruiting healthy volunteers with increased BMI and no cardiovascular risk factors for Group 2. Using our previously published LV-GLS for normal weight (-20.4±1.8%) and overweight non-diabetic patients (-19.0±2.2%)(4), calculated effect size=0.70 (sigma=2.0), β=0.80, α=0.05, a total of 100 patients will need to be recruited (25 in Group 1, 25 in Group 2, and 50 in Group 3).

**SUPPLEMENTAL RESULTS**

**Correlates of total LV-myoFat volume**

Supplementary Table 1 shows all the significant univariable and multivariable determinants of total LV-myoFat volume. Men had significantly higher total LV-myoFat volume compared to women (7.8±4.3 vs. 5.7±3.8mL, p=0.017). Increasing LV mass was associated with higher total LV-myocardial fat volume (r=0.773, p<0.001). On multivariable analysis, the presence of diabetes*BMI interaction term (standardized β=0.383, p<0.001), HOMA-IR (standardized β=0.211, p<0.001) and LV mass (standardized β=0.548, p<0.001) were independently associated with total LV-myoFat volume (model R=0.91). These 3 variables accounted for nearly 83% of the total variation in the data seen for total LV myo-Fat volume.

**Correlates of total myocardial interstitial volume**

Supplementary Table 2 shows all the significant univariable and multivariable determinants of total myocardial interstitial volume. Men had significantly higher total myocardial interstitial volume compared to women (28.9±8.1 vs. 21.5±5.6mL, p<0.001), and there was an extremely high correlation between total myocardial interstitial volume and LV mass (r=0.95, p<0.001). To identify independent correlates of total myocardial interstitial fibrosis, significant univariables (BMI, diabetes, the presence of diabetes*BMI interaction term, HOMA-IR, LV mass, and total LV-myoFat volume) were entered as covariates into the multiple linear regression model. On multivariable analysis, the independent determinants of total myocardial interstitial volume were total LV-myoFat volume (standardized β=0.219, p<0.001) and LV mass (standardized β=0.778, p<0.001) (model R=0.96). The results did not change when age and hypertension were forced into the model.

| Supplementary Table 1. Significant univariable and multivariable determinants of total left ventricular myocardial fat volume | | | | | | |
| --- | --- | --- | --- | --- | --- | --- |
|  | ***Univariable*** | | ***Multivariable Model 1*** | | ***Multivariable Model 2*** | |
| Variable | Standardized β | p value | Standardized β | p value | Standardized β | p value |
| Male gender | 0.238 | 0.017 |  |  |  |  |
| BMI | 0.627 | <0.001 | 0.177 | 0.002 |  |  |
| Presence of diabetes | 0.628 | <0.001 | 0.240 | <0.001 |  |  |
| Presence of diabetes* BMI interaction | 0.736 | <0.001 |  |  | 0.383 | <0.001 |
| Age | 0.381 | <0.001 |  |  |  |  |
| RPP | 0.337 | <0.001 |  |  |  |  |
| HbA1c | 0.502 | <0.001 |  |  |  |  |
| HOMA-IR | 0.601 | <0.001 | 0.230 | <0.001 | 0.211 | <0.001 |
| Plasma TG | 0.269 | <0.001 |  |  |  |  |
| LV mass | 0.551 | <0.001 | 0.556 | <0.001 | 0.548 | <0.001 |
| BMI: body mass index; HbA1c: glycated hemoglobin; HOMA-IR: homeostatic model assessment index of insulin resistance; LV: left ventricular; RPP: rate pressure product; TG: triglyceride.  Variables included in multivariable model 1: gender, BMI, presence of diabetes, age, RPP, HOMA-IR, plasma TG, LV mass  Variables included in multivariable model 2: model 1 + presence of diabetes*BMI interaction | | | | | | |

| Supplementary Table 2. Significant univariable and multivariable determinants of total myocardial interstitial volume | | | | |
| --- | --- | --- | --- | --- |
|  | ***Univariable*** | | ***Multivariable*** | |
| Variable | Standardized β | p value | Standardized β | p value |
| Male gender | 0.462 | <0.001 |  |  |
| BMI | 0.365 | 0.001 |  |  |
| Presence of diabetes | 0.364 | 0.001 |  |  |
| Presence of diabetes* BMI interaction | 0.465 | <0.001 |  |  |
| LV mass | 0.949 | <0.001 | 0.778 | <0.001 |
| HbA1c | 0.234 | 0/043 |  |  |
| HOMA-IR | 0.302 | 0.009 |  |  |
| Total LV-myoFat volume | 0.825 | <0.001 | 0.219 | <0.001 |
| BMI: body mass index; HbA1c: glycated hemoglobin; HOMA-IR: homeostatic model assessment index of insulin resistance; LV: left ventricular; LV-myoFat: left ventricular myocardial fat content; TG: triglyceride. | | | | |

| Supplementary Table 3. Significant univariable and multivariable determinants of left ventricular global longitudinal strain | | | | |
| --- | --- | --- | --- | --- |
|  | ***Univariable*** | | ***Multivariable Model*** | |
| Variable | Standardized β | p value | Standardized β | p value |
| Age | 0.437 | <0.001 |  |  |
| RPP | 0.467 | <0.001 | 0.331 | 0.001 |
| Plasma TG | 0.351 | <0.001 |  |  |
| LV mass | 0.510 | <0.001 |  |  |
| Total LV-myoFat volume | 0.664 | <0.001 |  |  |
| Total myocardial interstitial volume | 0.546 | <0.001 |  |  |
| Total LV-myoFat volume* Total myocardial interstitial volume interaction | 0.548 | <0.001 | 0.482 | <0.001 |
| BMI: body mass index; ECV: extracellular volume; LV: left ventricular; LV-myoFat: left ventricular myocardial fat content; RPP: rate pressure product; TG: triglyceride. | | | | |

Reference List

1. Münster M. SEED Haematology, Sysmex Educational Enhancement and Development. <https://www>.sysmex-europe.com/fileadmin/media/f100/SEED/Sysmex_SEED_The_Red_Blood_Cell_Indices pdf 2012.

2. Friedewald WT, Levy RI, Fredrickson DS. Estimation of the concentration of low-density lipoprotein cholesterol in plasma, without use of the preparative ultracentrifuge. Clin Chem 1972;18:499-502.

3. Faul F, Erdfelder E, Lang AG, Buchner A. G*Power 3: a flexible statistical power analysis program for the social, behavioral, and biomedical sciences. Behav Res Methods 2007;39:175-191.

4. Ng ACT, Prevedello F, Dolci G et al. Impact of Diabetes and Increasing Body Mass Index Category on Left Ventricular Systolic and Diastolic Function. J Am Soc Echocardiogr 2018;31:916-925.
